# The use and perception of support walkers for children with disabilities: a United Kingdom survey

**DOI:** 10.1186/s12887-020-02401-5

**Published:** 2020-11-18

**Authors:** Ciaran George, Wendy Levin, Jennifer M. Ryan

**Affiliations:** 1 College of Health, Medicine and Health Sciences, Brunel University London, London, UK; 2 Paediatric Physiotherapy Department, Royal Free London NHS Foundation Trust, London, UK; 3 Department of Public Health and Epidemiology, RCSI University of Medicine and Health Sciences, Dublin, Ireland; 4 Mary Seacole Building, Brunel University London, Kingston Lane, London, Uxbridge UB83PH UK

**Keywords:** Paediatric, Support walker, Disability

## Abstract

**Background:**

Support walkers are a type of assistive device that may enable non-ambulant children with disabilities to walk independently and promote improvements in bowel function, bone mineral density (BMD), mobility, independence, participation and social function. However, there is little evidence to support these benefits and there is a lack of research describing the use of support walkers in clinical practice. This study aimed to examine the use of support walkers for children with disabilities in clinical practice.

**Method:**

A survey was distributed via professional organisations, charities and schools associated with paediatric disabilities in the UK. Participants were recruited between January and March 2018. Populations of interest were those who prescribe support walkers to children with disabilities and those who work with children who use them.

**Results:**

In total, 125 people were included in the analysis; 107 responders prescribed support walkers and 18 responders worked with children who used support walkers. The population of children who use support walkers ranged from 6 months to 18 years and included children with cerebral palsy, chromosomal abnormalities and other medical conditions. Use of these devices was also reported in schools, at home and in the community for varying lengths of time. Numerous perceived benefits were noted, most frequent of which were increases in physical activity and enjoyment. By comparison, fewer perceived problems were identified but centred on lack of space and difficulty with transfers.

**Conclusions:**

This study provides insight into the use of support walkers in the UK, particularly surrounding current practices, which may help to improve consistency in clinical settings. Perceived benefits and problems may provide a basis for identification of appropriate outcome measures to monitor effectiveness. These results should also provide a basis for designing future studies to examine effectiveness of support walkers for paediatric disabilities.

**Supplementary Information:**

The online version contains supplementary material available at 10.1186/s12887-020-02401-5.

Children with disabilities, particularly those with severe motor impairments, participate in less physical activity and exhibit more sedentary behaviour than their typically developing peers [1, 2]. Barriers to participation in physical activity include stigma, negative attitudes, lack of motor skills and inaccessible environments [3, 4]. Assistive devices are one method used to enable children to overcome these barriers and hence increase physical activity.

Handheld walkers are commonly used to promote independent locomotion. However, these may not be feasible for children with severe mobility impairments who lack the motor control or strength to stand upright [5]. Support walkers are defined as any device that permits mobility for children who require more support than provided by handheld walkers, including some level of trunk, pelvic and/or head support [6]. Also known as ‘gait trainers’, there are many versions available with alterations in size, steering, seating and a range of accessories [6].

Support walkers may enable non-ambulant children the opportunity to walk independently. In a systematic review, several studies found positive changes across all dimensions of the International Classification of Functioning, Disability and Health [7], following use of support walkers including bowel function, bone mineral density (BMD), mobility, independence, participation and social function, with minimal adverse events [5]. However, the same systematic review suggested that the evidence was scarce, of low quality and inconsistent [5]. It was also noted that most of the current research focuses on children under 12 years of age with cerebral palsy [5], despite the potential of these devices in numerous other medical conditions [6]. Studies varied in terms of the protocol used and the outcomes assessed, thus limiting the conclusions that may be used to inform best practice.

While some studies have investigated the effectiveness of support walkers on a variety of outcomes, there is a lack of research examining how support walkers are currently used for children with disabilities in clinical practice. While one study has described the use of support walkers among children with disabilities in the United States [6], the study did not fully explore the perceived benefits, problems and reason to discontinue use of support walkers. Before further evaluating effectiveness of support walkers, information regarding the population who are prescribed support walkers, when and how they are used in routine clinical practice, and the perceived benefits and problems of using support walkers, is needed [8]. As such, this study aimed to examine the use of support walkers for children with disabilities in clinical practice. Specific questions were: (1) Which children use support walkers?; (2) Which support walkers are prescribed and which factors affect this decision?; (3) How are support walkers used?; (4) What are the perceived benefits and problems associated with using support walkers?

## Methodology

### Design

A cross-sectional survey design was used. Approval for the current research study was granted by Brunel University London Research Ethics Committee (Reference: 7627-A-Jan/2018–10,863-1). Completion of the survey indicated consent. All responses were anonymous.

### Participants

Participants were recruited over 10 weeks between January and March 2018. Professionals who prescribe support walkers, such as physiotherapists (i.e., prescribers), and those who work with children who use support walkers but do not prescribe them, such as teachers (i.e., non-prescribers) were invited to complete a survey. To be included in the study, prescribers must have prescribed a support walker to at least one child, aged 0–18 years. To be included in the study, non-prescribers must have worked with at least one child, aged 0–18 years who used, or currently uses a support walker. A support walker was defined as any device that permits mobility for children who require more support than provided by handheld walkers, including some level of trunk, pelvic and/or head support [6]. No restrictions were placed on type of disability due to the exploratory nature of this study.

Information about the study was shared with 33 Special Educational Needs (SEN) schools, 9 professional organisations and 10 charities for young people with disabilities in the UK. These were identified via an internet search. Emails were sent and replies were received from eight UK organisations and charities for children with disabilities (e.g., the Association of Paediatric Chartered Physiotherapists) and eight SEN schools. Gatekeepers who worked in each organisation (e.g. an administrator, a physiotherapist, a principal) distributed a brief description of the study and link to the information sheet and survey via email or social media. One school was provided with paper versions of the survey and information sheet to distribute at their request.

### Data collection

Separate surveys were developed for prescribers and non-prescribers following a literature review [5, 6, 9–12] and discussion with an experienced paediatric physiotherapist who prescribes support walkers. Both surveys included questions relating to characteristics of the children, normal use of the devices, and the perceived benefits and problems associated with their use. The penultimate draft of each survey was reviewed by an advisory panel, consisting of a physiotherapist and two teachers working in a SEN school, who assessed the questions for ease of interpretation, accuracy and comprehensiveness. Input from this panel prompted the following changes: the addition of responses to multiple questions, rephrasing of several questions, removal of questions that added confusion, and the addition of questions that required further expansion. At the start of each survey, participants were provided with the definition of a support walker, as stated previously, with 3 typical examples of support walkers.

The surveys contained 28 and 29 questions for the non-prescriber and prescriber surveys respectively, taking approximately 10–15 min to complete. The terms ‘Please tick all that apply’ or ‘Please select only one response’ followed the questions to maximise clarity. Most questions were close-ended, although there were three open-ended questions to permit greater exploration of opinions and depth of understanding. The open-ended questions were: “What, in your opinion, would be the ideal age to begin use of a support walker?”; “Please identify if there are any precautions for use of support walkers (e.g. supervision)” (Only present in the prescribers survey); “Any other comments?”

### Data analysis

Prior to data analysis, surveys in which responders reported working exclusively with adults (over 18 years old) were excluded. Descriptive statistics (mean, SD, range and percentages) were used to report data. If five or more participants provided the same response to a question where there was an option to select “other”, these responses formed a new category. Where a range of ages was given in response to the ‘age of introduction’ questions, the mean of this range was calculated and subsequently included in the calculation for mean of all responders. Responses to open questions were analysed using a simplistic form of content analysis, whereby similar responses were assigned a code and percentages of each code were calculated. Responses to the ‘any other comments’ question underwent a more detailed process of content analysis as described by Elo and Kyngäs [13], whereby important phrases were noted, matched to similar phrases and subsequently grouped into categories. The final stage, ‘interpretation’, was removed from this content analysis since the nature of inquiry did not permit knowledge of context or the ability to probe participants [14].

## Results

One hundred twenty-six surveys were returned; 107 from prescribers and 19 from non-prescribers. One non-prescriber survey was excluded as the responder reported only working with adults; thus, 18 were included in analysis. No response rates could be calculated as it was unknown how many eligible individuals received the survey.

### Responder demographics

Most prescribers were female (97.2%) and physiotherapists (98.1%). One occupational therapist and one therapy assistant also reported prescribing support walkers. Prescribers reported working across multiple settings including the community setting (80.4%), SEN schools (57.9%), mainstream schools (42.1%), outpatient settings (28.0%), inpatient settings (6.5%) and ‘other’ areas (3.7%). The occupation of non-prescribing professionals was more heterogeneous, comprising of physiotherapists (33.3%), occupational therapists (33.3%), teachers (22.2%), classroom assistants (5.6%) and other (5.6%). Prescribers and non-prescribers had worked with children using support walkers for a mean of 16.1 (range 2–35 years), and 11.7 (range 1–25 years) years, respectively.

### Characteristics of the children

Table 1 outlines the characteristics of children using support walkers as reported by prescribers and non-prescribers. Support walkers were most commonly reported to be prescribed and used by children between 2 and 12 years (Table 1). However, support walkers were prescribed and used by children of all ages (0–18 years), with three prescribers (2.8%) prescribing support walkers to children between 0 and 1 years (Table 1). Prescribers reported that the mean (standard deviation [SD]) age of introduction to support walkers was 3.6 (1.6) years. The youngest age of introduction, however, was reported by prescribers and non-prescribers as a mean (SD) of 2.4 (1.4) years (range; 1–14 years) and 2.9 (1.0) years (range; 1–5 years), respectively. When asked at what age children should ideally be introduced to support walkers, 77 prescribers responded giving a mean (SD) age of 2.5 (1.0) years (range 1–6 years). Thirty prescribers provided text responses which revealed that ideal age of introduction was dependent on the child’s condition (*n* = 20, 66.7%), more specifically their stage of development, cognition, motivation and motor ability. Five responders (16.7%) reported support walkers were often prescribed following recognition that a child was attempting to weight bear and step.

**Table 1:** Characteristics of the children who use support walkers as reported by prescribers and non-prescribers

	Prescribers (*n* = 107) *n* (%)	Non-prescribers (*n* = 18) *n* (%)
Age groups		
0–1 years	3 (2.8)	2 (11.1)
1–2 years	57 (53.3)	3 (16.7)
2–5 years	95 (88.8)	13 (72.2)
5–12 years	93 (86.9)	14 (77.8)
12–16 years	77 (72.0)	11 (61.1)
16–18 years	48 (44.9)	7 (38.9)
Medical Diagnosis		
Spastic CP	107 (100)	17 (94.4)
Dyskinetic CP	90 (84.1)	13 (72.2)
Ataxic CP	78 (72.9)	12 (66.7)
Chromosomal Abnormalities	83 (77.6)	9 (50.0)
Spina bifida	56 (52.3)	3 (16.7)
Muscular Dystrophy	53 (49.5)	6 (33.3)
Rett syndrome	49 (45.8)	3 (16.7)
Epilepsy	46 (43.0)	10 (55.6)
Spinal Cord Injury	41 (38.3)	2 (11.1)
Developmental Delay^a^	16 (15.0)	–
Other	15 (14.0)	1 (5.6)
Alternative form of mobility		
Wheelchair	98 (91.6)	16 (88.9)
Handheld walker	22 (20.6)	8 (44.4)
Nothing (independent walker)	16 (15.0)	2 (11.1)
Adult facilitated^a^	6 (5.6)	–
Other	12 (11.2)	5 (27.8)

Dash (−) indicates the items that were unavailable to that population

^a^represents a category that was created when enough responders reported it within the ‘other’ option

All prescribers reported prescribing support walkers to children with spastic cerebral palsy. Many prescribers also reported prescribing support walkers to children with dyskinetic CP, ataxic CP, chromosomal abnormalities and spina bifida (Table 1). Similarly, the majority of non-prescribers reported that children with spastic CP, dyskinetic CP, ataxic CP and chromosomal abnormalities used support walkers (Table 1). However, only 16.7% of non-prescribers reported that children with spina bifida used support walkers compared to 52.3% of prescribers (Table 1).

Approximately 90% of prescribers and non-prescribers reported that children using support walkers also used a wheelchair (Table 1). However, 20.6% of prescribers reported that children who used a support walker can walk with a handheld walker and 15.0% of prescribers reported that children who used a support walker can walk independently (Table 1).

### Prescription of support walkers

A child’s level of mobility was the most common factor affecting prescription of support walkers, as reported by 97.2% of prescribers (Table 2). A child’s tolerance of the support walker and current activity level were the second and third most reported determinants of support walker prescription (Table 2). Many prescribers noted that lack of head control (78.5%), pain (74.8%), and behavioural issues (57.0%) were contraindications to the prescription of support walkers (Table 2). Eighty percent of prescribers reported that the needs of the child affected the duration of use they prescribed, followed by clinical experience (76.6%), goals of physiotherapy (59.8%), evidence (26.2%), and local or national guidelines (9.3%) (Supplemental Table 1).

**Table 2:** Responses from prescribers regarding the factors affecting prescription and common contraindications

**Factors influencing prescription of support walkers**	**Prescribers (*n*** **= 107)** ***N*** **(%)**
A child’s mobility level	104 (97.2)
A child’s tolerance of the support walker	93 (86.9)
The activity level of the child	86 (80.4)
A child’s enjoyment	74 (69.2)
A child’s motor control	68 (63.6)
Gait pattern	63 (58.9)
Family support	60 (56.1)
Space to use and store the support walker	58 (54.2)
A child’s cognitive status	57 (53.3)
Muscle weakness	49 (45.8)
Medical needs of the child	39 (36.4)
A child’s balance	38 (35.5)
Cost of the support walker	27 (25.2)
Available evidence	23 (21.5)
Child’s aerobic endurance	22 (20.6)
Type of school a child attends	22 (20.6)
Other	8 (7.5)
**Common contraindications to the use of support walkers**	**Prescribers (*n*** **= 107)** ***N*** **(%)**
Lack of head control	84 (78.5)
Pain experienced in the walker	80 (74.8)
Behaviour issues	61 (57.0)
Current hip dislocation	39 (36.4)
Cognitive impairment	35 (32.7)
Muscle contractures	32 (29.9)
Skeletal deformities	23 (21.5)
The weight of the child	22 (20.6)
Lack of trunk control	19 (17.8)
Visual impairment	18 (16.8)
Epilepsy	17 (15.9)
Reduced bone mineral density	16 (15.0)
The height of the child	12 (11.2)
Risk of hip dislocation	11 (10.3)
Other	7 (6.5)

In total, 21 support walkers were identified by prescribers (Supplemental Table 2). Most commonly prescribed support walkers were the Rifton Pacer (88.8%), Ormesa Grillo (39.3%) and Buddy Roamer (35.5%) (Supplemental Table 2). When choosing which support walker to prescribe, prescribers most commonly reported that clinical experience (91.6%), needs of the child (89.7%) and goals of physiotherapy (72.9%) affected their decision (Supplemental Table 3). The inclusion of parents (98.1%) and the child (82.2%) within this decision was also reported by most prescribers (Supplemental Table 3).

### Use of support walkers

The environments in which prescribers reported support walkers are used included: at home (88.8%), in schools (94.4%) and in the community (69.2%) (Supplemental Table 4). Similarly, non-prescribers reported support walkers were used in schools (100%), at home (72.2%) and in the community (38.9%) (Supplemental Table 4).

When asked how long prescribers would recommend using the support walker per day, 49.5% suggested ‘as much as able’ (Supplemental Table 5). Others recommended either: 10–30 min (5.6%); 30–60 min (31.8%), 1–2 h (11.2%) or 2–5 h (1.9%) (Supplemental Table 5). In contrast, non-prescribers reported that support walkers were used for less than 10 min (5.9%), 10–30 min (35.3%), 30–60 min (35.3%), 1–2 h (17.6%) or all day (5.9%) (Supplemental Table 5). The perceived reasons for discrepancies between prescribed and actual use are given in Table 3. Most prescribers suggested a lack of staff to help the child (88.8%) and/or a lack of space to use the walker (72.9%) prevented use (Table 3). However, among non-prescribers, 39% did not know which factors prevented the duration being achieved and 39% reported that the child simply preferred another means of mobility (Table 3).

**Table 3:** Reasons why ‘actual’ duration may be less than the prescribed duration

	Prescribers (*n* = 107), *N* (%)	Non-prescribers (*n* = 18), *N* (%)
Lack of staff to help the child	95 (88.8)	6 (33.3)
Lack of space to use the walker	78 (72.9)	4 (22.2)
Child prefers other means of mobility	36 (33.6)	7 (38.9)
The walker causes the child pain	19 (17.8)	2 (11.1)
The child doesn’t like the look	4 (3.7)	0 (0.0)
Boredom	20 (18.7)	2 (11.1)
The walker is uncomfortable to use	22 (20.6)	0 (0.0)
Doesn’t like different position	8 (7.5)	0 (0.0)
They are shared between children	16 (15.0)	4 (22.2)
Fatigue^a^	6 (5.6)	–
Other	15 (14.0)	3 (16.7)
Don’t know	–	7 (38.9)

Dash (−) indicates the items that were unavailable to that population

^a^represents a category that was created when 5 or more similar responses were given in the ‘other’ option

Ninety prescribers (84.1%) provided a response to question “Please identify if there are any precautions for use of support walkers”. The requirement for supervision was most commonly reported (*n* = 73, 68.2%). The second most frequently reported precaution was the need for a suitable environment including a suitable surface, adequate space and distance from other children (*n* = 48; 53.3%). Other precautions to consider were the need for competent or trained staff and parents (*n* = 25; 27.8%) and the presence of other medical conditions, particularly those with epilepsy (*n* = 8; 8.9%).

Before use of these devices is discontinued by a child, 42.1% of prescribers reported that they are used for 5–10 years (Supplemental Table 6). Others reported that they were used for less than 1 year (1.9%), 1–2 years (2.8%), 2–5 years (34.6%), and over 10 years (18.7%) (Supplemental Table 6). Non prescribers reported use for 2–5 years (50%), 5–10 years (27.8%), over 10 years (11.1%), 1–2 years (5.6%) or less than 1 year (5.6%) (Supplemental Table 6).

Approximately 75 % of prescribers suggested that between 0 and 20% of children who use support walkers show progression in walking ability, such that they no longer require the support walker (Supplemental Table 7). When asked what affects a child’s chance of walking progression, most prescribers reported that it was due to: the child’s condition (96.3%), original level of head or trunk control (79.4%), frequency of use (51.4%), age of introduction (21.5%), motivation (10.3%) and cognitive ability (6.5%) (Supplemental Table 7).

Table 4 reports the reasons children discontinue use of their support walker. Progression of their condition, such that they are no longer able to use support walkers was most frequently reported by prescribers (79.4%) and non-prescribers (72.2%) (Table 4). Both groups also reported that discontinuation occurred because children prefer other means of mobility or progress their walking ability (Table 4).

**Table 4:** Responses given for the reason children discontinue using support walkers

	Prescribers (*n* = 107), *N* (%)	Non-prescribers (*n* = 18), *N* (%)
Progression of condition (i.e. unable to use the SW)	85 (79.4)	13 (72.2)
Other means of mobility are preferred	50 (46.7)	11 (61.1)
Progression of walking ability	46 (43.0)	9 (50.0)
Frustration with the SW	40 (37.4)	7 (38.9)
Not enough space to use the SW	37 (34.6)	5 (27.8)
Safety reasons regarding the size of the child	36 (33.6)	6 (33.3)
Fractures or other medical complications	25 (23.4)	4 (22.2)
Other	12 (11.2)	1 (5.6)
Left the school that provided the SW	8 (7.5)	2 (11.1)
Negative social perceptions	6 (5.6)	1 (5.6)
Boredom	6 (5.6)	2 (11.1)

*SW* Support Walkers

### Perceived benefits and problems

Table 5 shows the perceived benefits of using support walkers. The three most reported perceived benefits by prescribers were increasing physical activity time (98.1%), enjoyment (94.4%), and increasing participation (90.7%). Increased independence (94.4%), increased time being physically active (94.4%), and enjoyment (83.3%) were most reported perceived benefits by non-prescribers (Table 5). However, many more perceived benefits were reported by over half of prescribers and non-prescribers, including improved muscle strength, improved motor abilities, increased peer and family interaction, improved respiratory function and increased bone mineral density (Table 5).

**Table 5:** Perceived Benefits of using support walkers as perceived by all responders

	Prescribers (*n* = 107) *n* (%)	Non-Prescribers (*n* = 18) *n* (%)
Increase time being physically active	105 (98.1)	17 (94.4)
Enjoyment	101 (94.4)	15 (83.3)
Increase participation in everyday life	97 (90.7)	15 (83.3)
Provides different opportunities to access their environment	95 (88.8)	13 (72.2)
Increase independence	92 (86.0)	17 (94.4)
Increased confidence	91 (85.0)	13 (72.2)
Improved muscle strength	91 (85.0)	14 (77.8)
Provides a change of position	91 (85.0)	14 (77.8)
Improved motor abilities (i.e. walking)	89 (83.2)	12 (66.7)
Increased peer and family interaction	89 (83.2)	14 (77.8)
Improved respiratory function	73 (68.2)	8 (44.4)
Increase bone mineral density	67 (62.6)	9 (50.0)
Improved head and trunk control	65 (60.7)	8 (44.4)
Improved bladder and bowel function	58 (54.2)	8 (44.4)
Improved problem solving (i.e. navigation)	54 (50.5)	9 (50.0)
Prevents muscle wasting	50 (46.7)	8 (44.4)
Improved communication	36 (33.6)	7 (38.9)
Improved cognition	22 (20.6)	3 (16.7)
Improved vision	9 (8.4)	3 (16.7)
Other	4 (3.7)	2 (11.1)

The perceived problems experienced with support walkers are shown in Table 6. Lack of space was the most commonly reported problem among prescribers (79.4%) (Table 6). Other perceived problems reported by prescribers included: getting in or out of the walker (67.3%), and lack of accessibility (64.5%) (Table 6). Poor manoeuvrability was the most commonly reported problem perceived by non-prescribers (66.7%) (Table 6).

**Table 6:** Perceived problems with support walkers as perceived by all responders

	Prescribers (*n* = 107), *N* (%)	Non-prescribers (*n* = 18), *N* (%)
Not enough space	85 (79.4)	10 (55.6)
Getting the child in or out of the SW	72 (67.3)	9 (50.0)
Lack of accessibility	69 (64.5)	10 (55.6)
The time required to transfer in and out of the SW	50 (46.7)	3 (16.7)
Trouble moving on difference surfaces	47 (43.9)	10 (55.6)
Time to use the SW	45 (42.1)	4 (22.2)
Poor manoeuvrability	37 (34.6)	12 (66.7)
Lack of knowledge about SW	21 (19.6)	3 (16.7)
Problems adjusting the walker if shared	16 (15.0)	2 (11.1)
Risk of tipping	12 (11.2)	5 (27.8)
Negative social perceptions	8 (7.5)	3 (16.7)
Other	7 (6.5)	2 (11.1)

*SW* Support Walkers

#### ‘Any other comments?’

Thirty-three participants (29 prescribers and 4 non-prescribers) provided a response to the question ‘any other comments’. Where quotations are reported below, the responder’s individual identification (ID) number follows.

### Category 1: the age to introduce support walker

This category details two conflicting points of view regarding the age that support walkers should be introduced. The first is that support walkers should be introduced early as this may precipitate independent walking. Conversely, others believed that support walkers should only be introduced after no further gain in walking ability is expected. The contrasting points of view are exemplified below.

“we strive to get children walking as soon as they tolerate supported standing or start nursery when peers are exploring areas in standing … many go on to independent walking without aids. An early approach to walking puts steps in place for the child to progress quicker and reduced frustration at not being able to get about.” (Participant 38)

“[I] generally don’t prescribe them until later when it becomes evident that the child’s walking potential has been met and only then for enjoyment and [the] other factors mentioned” (Participant 17)

### Category 2: lack of funding

Comments highlighting issues acquiring funding for support walkers were common. For example,

“it is frustrating that they mostly have to be privately funded or by seeking charity funds” (Participant 103)

Some prescribers also noted that even when funding was available certain criteria must be met, such that they may only be prescribed if the child is expected to gain the ability to walk, as in the next quote.

“our manager will not allow NHS funding for a supportive walker unless it is likely to lead to that child becoming an independent walker” (Participant 99)

### Category 3: perceived benefits

Several prescribers remarked that support walkers are primarily for non-ambulant children, although a wide range of children may use them. The quotations below exemplify this:

“In my experience walkers give children with otherwise no independent mobility a change of position, exercise and mobility which they love” (Participant 57)

“Some early [developmental] delay children we also put in as a way of gaining weight bearing but they will not be long term users and progress to independent walking.” (Participant 89)

## Discussion

This study aimed to examine the use of support walkers for children with disabilities in clinical practice in the UK. The findings highlight that support walkers are prescribed to children with a variety of medical conditions including cerebral palsy, chromosomal abnormalities and spina bifida. Mobility level, tolerance and activity level of the child were the most reported factors affecting prescription of support walkers which were subsequently used for differing lengths of time per day. Many perceived benefits, including increasing physical activity, enjoyment and participation were reported. A substantial gap in the literature on support walkers exists, making comparisons to previous research difficult.

Below is a discussion of the findings in relation to each research question.

### Which children use support walkers?

While prescribers and non-prescribers report that most children who use support walkers have cerebral palsy, these findings highlight that support walkers are prescribed to children with a substantial diversity of conditions. Children with chromosomal abnormalities were the third most frequently reported group that support walkers were prescribed to, even though no previous research examining the effects of support walkers have included this population.

This study found that some children are prescribed support walkers from as young as one-year-old. However, children below the age of 3½ years old are frequently excluded from participating in studies examining effectiveness of support walkers [9, 10]. Although, an average of 2½ years old was deemed ‘ideal’, this is far behind the 12.1 months at which typically developing children begin walking independently [15]. It may be that earlier introduction encourages motor development and consequently psychological and cognitive development [16]. It was clear that earlier use was encouraged by some prescribers who noted a potential to progress to less supportive walking aids as development occurs. However, contrary to that opinion, others indicated that they would introduce support walkers only when no further improvement in walking was seen. These diverse opinions among prescribers may explain why there was substantial variation in responses from prescribers with regards to the “ideal” age at which to introduce support walkers, with a range between 1 and 5 years. Many responders reported that the ‘ideal’ age is specific to the individual and depends upon the needs of the child. It may be that differences in the perceived benefits of support walkers among prescribers may contribute to diversity of opinion regarding the age at which to introduce them.

### Which support walkers are prescribed and which factors affect this decision?

Several types of support walkers were reportedly used, most of which currently have no research investigating their effectiveness. However, approximately a quarter of responders suggested that the available evidence influenced their decision. A child’s mobility level and motor control were commonly reported to influence prescription of support walkers. Previous research examining effectiveness has commonly excluded children of higher motor function [9, 11]. However, prescribers in this study reported that support walkers may be used as a bridge to independent walking, with improved walking ability being a reason for discontinuation. This is in agreement with findings from previous studies [11, 17].

### How are support walkers used?

Most responders reported that children continue using support walkers for 5–10 years, far exceeding the duration of follow-up in previous research, which ranged from 3 months to 3 years [6, 9–11]. Although interventions imposed time restrictions for obvious reasons, Low et al. [6] indicated that they are most commonly used for less than 6 months. Since no explanation was given by Low et al. [6] for discontinuation, it can only be speculated that there exists differences in the prescription and use of support walkers between the UK and USA.

Deterioration of a child’s condition was identified in this study as the most reported reason for discontinuation. The progressive nature of some conditions (e.g. muscular dystrophy) or negative changes that occur during growth spurts (e.g. scoliosis and hip problems) will likely make walking difficult or impossible for these children [18]. The resulting pain or increased energy requirements may explain why a preference for another form of mobility (e.g. wheelchairs) may subsequently arise [19].

Almost half of prescribers reported support walkers should be used as much as able, suggesting that longer use is encouraged. However, both prescribers and non-prescribers also noted that a lack of staff limited the duration of use. Huang et al. [20] remarked that teachers are rarely able to encourage the use of assistive devices, as they are preoccupied with large class sizes and academic success of every child. As such, the requirement for supervision, as noted by the majority of responders, is likely unfeasible in most educational settings including SEN provision, at least for a prolonged period. Lack of space was also noted to affect how long support walkers are used, as well as represent a precaution to be considered when prescribing support walkers.

### What are the perceived benefits and problems associated with using support walkers?

Responders perceived that support walkers have numerous benefits. The most commonly reported perceived benefits were increased physical activity, participation and enjoyment. Fewer prescribers and non-prescribers reported physiological changes associated with use of support walkers, including bone mineral density and bladder and bowel function. Despite never being examined as an outcome in studies of effectiveness of support walkers, increasing the amount of time a child is physically active was the most reported benefit by prescribers and non-prescribers. For a group that commonly exhibits less physical activity, support walkers may therefore promote participation in physical activity and associated health benefits, such as reduced pain, fatigue and osteoporosis among children with disabilities [21]. In addition, partaking in physical activity may explain subsequent increases in confidence and self-esteem, which were noted as perceived benefits by the majority of responders and in previous research [12, 22].

The perception that support walkers increased participation in everyday life for children with disabilities was commonly reported. This may relate to many tasks encountered during the typical day, from education to play. Being upright affords children different opportunities to experience their surroundings, which was frequently reported in this research. McKeever et al. [12] found that parents thought an altered perception of the environment, which arose from being upright, increases participation, as well as interaction with peers and family members. Social function, however, showed no change in two previous studies of the effects of support walkers [9, 10]. Researchers have concluded that outcome measures available to assess participation may not adequately do so, which may explain the lack of support beyond these subjective accounts [23]. In addition to potential benefits on development and health, participation in leisure activities and greater enjoyment has been shown to increase a child’s quality of life (QOL) [24]. However, the effect of using support walkers on quality of life has not been examined and should be considered as an outcome in future studies of effectiveness.

Lack of space was perceived as a problem by many prescribers and non-prescribers in this study. Huang et al. [25] reported that a lack of space to move around or store big devices, like support walkers, led many children to abandon use of assistive devices at home. As one of the biggest assistive devices, it is not difficult to imagine why space, manoeuvrability and accessibility were frequently reported issues. Many prescribers and non-prescribers also perceived that transferring a child to the support walker, and the time required to do so, was a problem when using support walkers.

Instability and reports of support walkers tipping over were noted in previous literature when using a particular support walker (David Hart walking orthosis), particularly when on uneven surfaces outside [10]. Almost half of the prescribers and non-prescribers in this study also stated that using support walkers on different surfaces was problematic. Despite this, use in the community was reported by over half of prescribers and non-prescribers. Hence, rather than limiting use, the requirement for constant supervision, which was noted as a precaution by prescribers, may mitigate these risks.

### Limitations

A relatively small sample size and the inability to calculate response rates are perhaps the most important limitations associated with this study, especially for the non-prescribing group. The sample of convenience may have also introduced selection bias. In addition, despite the use of open-ended questions, the survey design prevented the ability to probe responders and thereby lack the conceptual richness and depth of knowledge. This study is also limited in that these results only provide a snapshot and do not adequately describe the process of prescription.

This study describes the paediatric populations who use support walkers, which may enable clinicians to consider their current practice, especially regarding the use of support walkers among children with medical conditions, such as chromosomal abnormalities, where no previous research exists. Identification of common contraindications or precautions for use may also support the clinical decision process when prescribing support walkers. Additionally, identification of common problems may enable individuals to put in place strategies to overcome these common barriers before prescribing support walkers. Prescribers should also take note of the perceived benefits, as they may be used to support an individual’s decision to use support walkers as well as provide a list from which appropriate outcome measures may be selected to monitor progress. The findings from this study may also provide a basis for future research. Randomised controlled trials are needed to establish the effectiveness of support walkers. These findings may support researchers to define the population, identify appropriate outcomes, and determine an appropriate duration of follow-up in future trials. Investigation of differences in outcomes between age groups or conditions may also promote clearer decisions surrounding the prescription of support walkers.

## Conclusion

In summary, this study provides an insight into the current use and perception of support walkers for children with disabilities in the UK. Findings suggest that the population of children that use support walkers is far more diverse than previously included in studies of effectiveness. There is large variation in current clinical practice, including the age at which support walkers are prescribed, the functional mobility of children who are prescribed support walkers, and the duration for which they are used. Numerous perceived benefits were reported, including increased participation, physical activity and enjoyment. The effectiveness of support walkers on many of these outcomes has not been previously examined. These findings should represent the first step to improve consistency in clinical practice and aid the clinical decision-making process. However, further research, with these findings in mind, must be conducted so that clinicians may deliver evidence-based practice.

## Supplementary Information

**Additional file 1: Supplemental Table 1.** Prescribers reasoning behind a given duration of use. **Supplemental Table 2.** Types of support walkers used among prescribers and non-prescribers. **Supplemental Table 3.** Responses from prescribers regarding the factors that influence which support walker is prescribed and the individuals involved in this decision. **Supplemental Table 4.** The environments in which support walkers are used, as reported by prescribers and non-prescribers. **Supplemental Table 5.** Prescribed and actual daily duration of support walker use, as reported by prescribers and non-prescribers. **Supplemental Table 6.** The duration support walkers are used for within a child’s lifespan, as reported by prescribers and non-prescribers. **Supplemental Table 7.** Percentage of children for whom their walking ability progresses (e.g. handheld or independent walking) and the factors which affect this.

**Additional file 2. **Example of a support walker

## Data Availability

The dataset analysed during the current study is available from the corresponding author on reasonable request.

## Abbreviations

QOL: Quality of life
SEN: Special Educational Needs
GMFCS: Gross motor function classification system
UK: United Kingdom
USA: United states of America
NHS: National Health Service
SW: Support walker
CP: Cerebral Palsy
SD: Standard Deviation
BMD: Bone mineral Density
