# Supplementary material for: The use and perception of support walkers for children with disabilities: a United Kingdom survey
Source: BMC Pediatr. 2020 Nov 18;20:528. doi: 10.1186/s12887-020-02401-5 (PMC7672809; doi:10.1186/s12887-020-02401-5)
Supplement: Supplementary file 1 — Additional file 1: Supplemental Table 1. Prescribers reasoning behind a given duration of use. Supplemental Table 2. Types of support walkers used among prescribers and non-prescribers. Supplemental Table 3. Responses from prescribers regarding the factors that influence which support walker is prescribed and the individuals involved in this decision. Supplemental Table 4. The environments in which support walkers are used, as reported by prescribers and non-prescribers. Supplemental Table 5. Prescribed and actual daily duration of support walker use, as reported by prescribers and non-prescribers. Supplemental Table 6. The duration support walkers are used for within a child’s lifespan, as reported by prescribers and non-prescribers. Supplemental Table 7. Percentage of children for whom their walking ability progresses (e.g. handheld or independent walking) and the factors which affect this. [file 12887_2020_2401_MOESM1_ESM.docx]

Appendix: Supplemental Tables.

Supplemental table 1: Prescribers reasoning behind a given duration of use

|  | Prescribers (n=107),  N (%) |  |
| --- | --- | --- |
| Reasons for the ‘prescribed duration’ | | |
| Needs of the child | 86 (80.4) | |
| Clinical experience | 82 (76.6) | |
| Goals of physiotherapy | 64 (59.8) | |
| Evidence | 28 (26.2) | |
| Other | 12 (11.2) | |
| Local or national guidelines | 10 (9.3) | |

*Dash (-) indicates the items that were unavailable to that population
*represents a category that was created when enough respondents reported it within the ‘other’ option.*

Supplemental table 2: Types of support walkers used among prescribers and non-prescribers

|  | **Prescribers (n=107),**  **N (%)** | **Non-Prescribers (n=18),**  **N (%)** |
| --- | --- | --- |
| Rifton Pacer Gait Trainer | 95 (88.8) | 14 (77.8) |
| Ormesa Grillo Walker | 42 (39.3) | 2 (11.1) |
| Buddy Roamer | 38 (35.5) | 1 (5.6) |
| Pony Gait Trainer | 36 (33.6) | 8 (44.4) |
| Meywalker | 35 (32.7) | 3 (16.7) |
| Broncho Walker | 30 (28.0) | 6 (33.3) |
| Lecky Kidwalk* | 21 (19.6) | 2 (11.1) |
| Mullholland Walkabout | 19 (17.8) | 4 (22.2) |
| R82 Mustang Walker* | 18 (16.8) | 2 (11.1) |
| DHWO | 10 (9.3) | 2 (11.1) |
| Kaye Walker* | 9 (8.4) | 1 (5.6) |
| Ormesa Dynamic Walker | 6 (5.6) | 2 (11.1) |
| Mowego Gait Trainer | 2 (1.9) | 0 (0.0) |
| Gaitmaster Gait Trainer | 1 (0.9) | 1 (5.6) |
| Other | 14 (13.1) | 2 (11.1) |

*Abbreviations: DHWO, David Hart Walking Orthosis
Dash (-) indicates the items that were unavailable to that population
*represents a category that was created when enough respondents reported it within the ‘other’ option.*

| Factors that informs the choice of a given support walker | N (%) |
| --- | --- |
| Clinical experience | 98 (91.6) |
| Needs of the child | 96 (89.7) |
| Goals of physiotherapy | 78 (72.9) |
| Availability of the support walkers | 60 (56.1) |
| Size (height/weight) of the child | 56 (52.3) |
| Evidence | 45 (42.1) |
| Funding | 44 (41.1) |
| Availability of accessories | 42 (39.3) |
| Seating available on the support walker | 8 (7.5) |
| Other | 7 (6.5) |
| Local or national guidelines | 6 (5.6) |
| Who else is involved in the decision | **N (%)** |
| Parents | 105 (98.1) |
| Child | 88 (82.2) |
| Teacher | 30 (28.0) |
| Occupational therapist | 14 (13.1) |
| Physiotherapist* | 8 (7.5) |
| Only me | 3 (2.8) |
| Other | 3 (2.8) |

Supplemental table 3: Responses from prescribers (n=107) regarding the factors that influence which support walker is prescribed and the individuals involved in this decision

**represents a category that was created when enough respondents reported it within the ‘other’ option*

Supplemental table 4: The environments in which support walkers are used, as reported by prescribers and non-prescribers

|  | Prescribers (n=107),  N (%) | Non-prescribers (n=18),  N (%) |
| --- | --- | --- |
| School | 101 (94.4) | 18 (100.0) |
| Home | 95 (88.8) | 13 (72.2) |
| Outside in community | 74 (69.2) | 7 (38.9) |
| Other | 14 (13.1) | 0 (0.0) |

Supplemental table 5: Prescribed and actual daily duration of support walker use, as reported by prescribers and non-prescribers

|  | Prescribers (n=107),  N (%) | Non-prescribers (n=17), N (%) | |
| --- | --- | --- | --- |
| Duration | Prescribed duration | Actual duration |  |
| Less than 10 minutes | 0 (0.0) | 1 (5.9) |  |
| 10-30 minutes | 6 (5.6) | 6 (35.3) |  |
| 30-60 minutes | 34 (31.8) | 6 (35.3) |  |
| 1-2 hours | 12 (11.2) | 3 (17.6) |  |
| 2-5 hours | 2 (1.9) | 0 (0.0) |  |
| All day | 0 (0.0) | 1 (5.9) |  |
| As much as able | 53 (49.5) | - |  |

*Dash (-) indicates the items that were unavailable to that population
Non-prescribers column: n=17 as one non-prescriber did not answer this question*

Supplemental table 6: The duration support walkers are used for within a child's lifespan, as reported by prescribers and non-prescribers

|  | Prescribers (n=107),  N (%) | Non-prescribers (n=18),  N (%) |
| --- | --- | --- |
| <1 year | 2 (1.9) | 1 (5.6) |
| 1-2 year | 3 (2.8) | 1 (5.6) |
| 2-5 years | 37 (34.6) | 9 (50.0) |
| 5-10 years | 45 (42.1) | 5 (27.8) |
| >10 years | 20 (18.7) | 2 (11.1) |

Supplemental table 7: Percentage of children for whom their walking ability progresses (e.g. handheld or independent walking) and the factors which affect this

|  | Prescribers (n=107),  N (%) | Non-prescribers (n=18),  N (%) |
| --- | --- | --- |
| Children who progress their walking ability |  |  |
| 0% | 5 (4.7) | 0 (0.0) |
| 1-10% | 40 (37.4) | 5 (27.8) |
| 11-20% | 35 (32.7) | 1 (5.6) |
| 20-40% | 15 (14.0) | 0 (0.0) |
| 40-60% | 7 (6.5) | 1 (5.6) |
| 60-80% | 4 (3.7) | 0 (0.0) |
| 80-90% | 1 (0.9) | 0 (0.0) |
| 90-100% | 0 (0.0) | 0 (0.0) |
| I don't know | - | 11 (61.1) |
| Factors affecting a child’s ability to become independent | |  |
| Child's condition | 103 (96.3) | 18 (100.0) |
| Frequency of use | 55 (51.4) | 10 (55.6) |
| Age the support walker was introduced | 23 (21.5) | 4 (22.2) |
| Original level of head and trunk control | 85 (79.4) | 11 (61.1) |
| Motivation* | 11 (10.3) | - |
| Cognitive ability* | 7 (6.5) | - |
| Other | 16 (15.0) | 4 (22.2) |

*Dash (-) indicates the items that were unavailable to that population
*represents a category that was created when enough respondents reported it within the ‘other’ option.*
