# Supplementary figures and images for: The use and perception of support walkers for children with disabilities: a United Kingdom survey
Source: BMC Pediatr. 2020 Nov 18;20:528. doi: 10.1186/s12887-020-02401-5 (PMC7672809; doi:10.1186/s12887-020-02401-5)

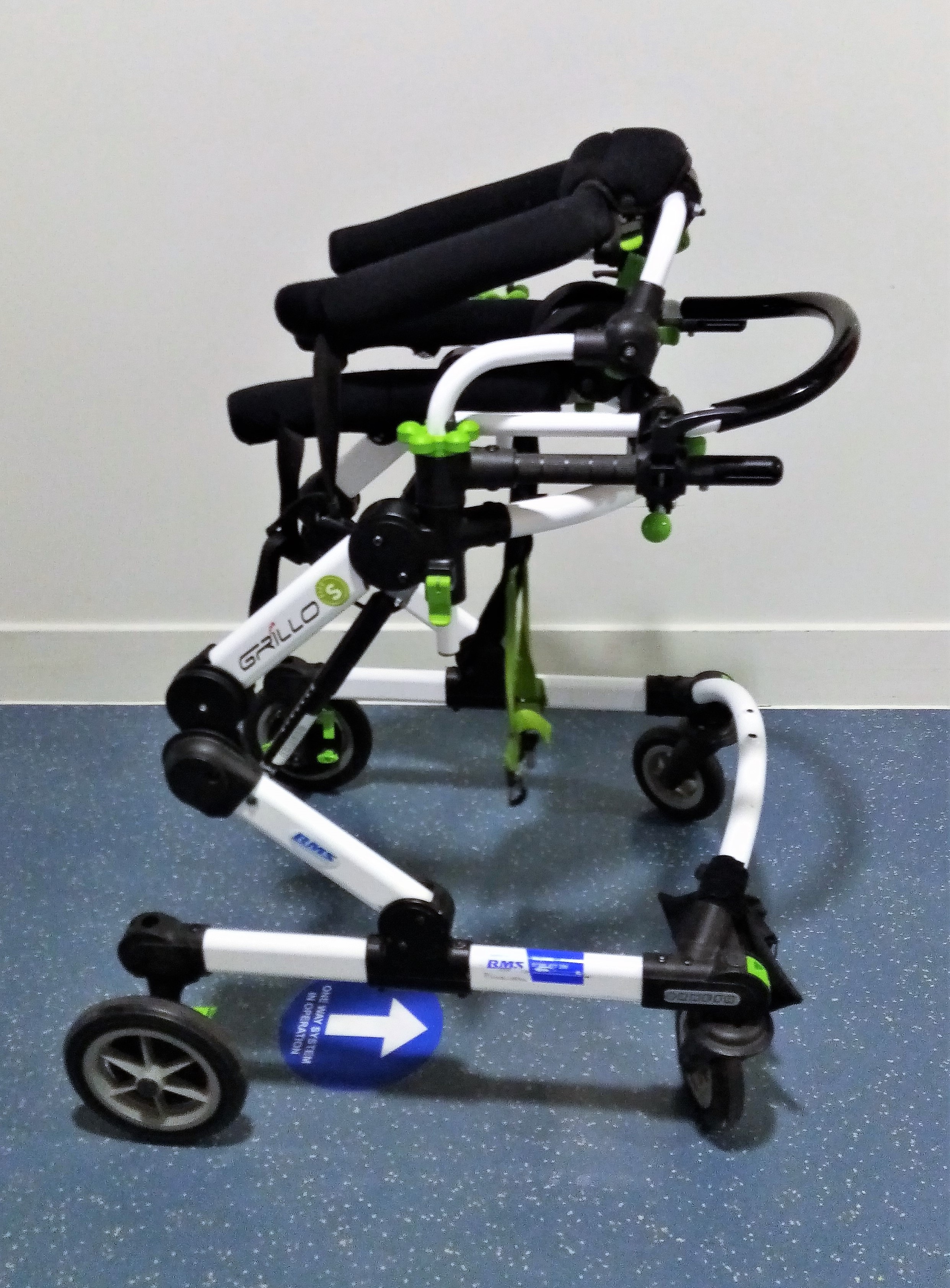

Supplement: Supplementary file 2 — Additional file 2. Example of a support walker [file 12887_2020_2401_MOESM2_ESM.jpg]
